# Clinical Factors of Delayed Perforation after Endoscopic Submucosal Dissection for Gastric Neoplasms

**DOI:** 10.1155/2017/7404613

**Published:** 2017-08-15

**Authors:** Yoshinobu Yamamoto, Hogara Nishisaki, Hideki Sakai, Nagahiro Tokuyama, Hiroaki Sawai, Aya Sakai, Takuya Mimura, Saeko Kushida, Hidetaka Tsumura, Takeshi Sakamoto, Ikuya Miki, Masahiro Tsuda, Hideto Inokuchi

**Affiliations:** ^1^Department of Gastroenterology, Hyogo Cancer Center, Hyogo 673-8558, Japan; ^2^Department of Internal Medicine, Kaibara hospital, Hyogo 669-3395, Japan

## Abstract

**Background:**

Delayed perforation is a rare but severe complication of endoscopic submucosal dissection (ESD) for early gastric neoplasm (EGN). The aim of this study was to clarify clinical factors related to delayed perforation after ESD.

**Methods:**

A total of 1158 consecutive patients with 1199 EGNs underwent ESD at our hospital between January 2000 and December 2015. Univariate analysis was used to identify clinicopathological factors related to delayed perforation. Moreover, duration of cautery needed for hemostasis was measured by comparison between perforated and nonperforated points in patients with delayed perforation.

**Results:**

Delayed perforation occurred in 5 of 1158 consecutive patients with 1199 EGNs who underwent ESD (0.42%). All cases were diagnosed within 24 h after ESD and recovered with conservative management. On univariate analysis, location in the upper stomach was the factor most significantly associated with delayed perforation (*P* < 0.01). Duration of cautery needed for hemostasis was significantly longer at perforated points (9 s) than at nonperforated points (3.5 s) in five patients.

**Conclusions:**

Location in the upper stomach was the risk factor most prominently associated with delayed perforation after ESD for EGNs. In addition, delayed perforation appears associated with excessive electrocautery for hemostasis.

## 1. Introduction

Endoscopic submucosal dissection (ESD) has become widespread as a treatment for early gastric neoplasms (EGNs) with a negligible risk of lymph node metastasis, such as early gastric cancer (EGC) and adenoma [1]. Compared with conventional endoscopic mucosal resection, ESD offers advantages of high curability without local recurrence. The major complications of gastric ESD are bleeding and perforation [2, 3]. Most perforations occur during the ESD procedures, with rates reportedly ranging from 1.2% to 9.6% [2–9]. Delayed perforation occurs after completion of ESD, even when perforation is not detected during the ESD procedure. Some studies have reported rates of delayed perforation of 0.06–0.45% after gastric ESD [3, 10–13]. Several case reports have also described delayed perforation after ESD for EGC [14–18]. The mechanism underlying small delayed perforation is considered to be excessive electrocautery for hemostasis [10, 11, 13–15, 17]. The correlation between duration of cautery and delayed perforation has not previously been reported. This appears to represent the first report to confirm a statistical correlation between duration of cautery and delayed perforation. Clinical risk factors and methods for the management of delayed perforation after ESD for EGN are described in this report.

## 2. Materials and Methods

### 2.1. Patients

A total of 1158 consecutive patients with 1199 EGNs underwent ESD at the Hyogo Cancer Center between January 2000 and December 2015. All lesions were diagnosed as gastric adenoma or adenocarcinoma. The indication for treatment of EGNs was based on the expanded indications accepted by the Japanese Gastric Cancer Association [19]. These expanded indications included lesions such as (i) differentiated intramucosal cancer without ulcers, irrespective of tumor size; (ii) differentiated intramucosal cancer, <3 cm in size, with ulceration; (iii) differentiated minute (<500 *μ*m from muscularis mucosae) submucosal invasive cancer, <3 cm in size; and (iv) undifferentiated intramucosal cancer without ulcer, <2 cm in size. Duration of cautery needed for hemostasis was measured by reviewing the video of the ESD procedure. While the bleeding point is cauterized by the hemostatic forceps, small bubbles, whitening of tissue, and steam are observed. Duration of cautery was defined the total time of cautery in which such above findings were observed. Three experts who performed gastric ESD more than 200 cases identified perforated and nonperforated points by reviewing the video and pictures of ESD procedure in contrast to the pictures with delayed perforation.

### 2.2. ESD Procedure

ESD procedures were performed as previously reported [20]. A high-frequency generator (ICC350 or VIO300D; ERBE, Tubingen, Germany) was used. Electrosurgical devices used in ESD were mainly the insulated-tip knife 2 (IT2, KD-610L or KD-611; Olympus, Tokyo, Japan) or the Flush knife BT (DK2618JB20; Fujifilm Medical, Saitama, Japan). The models used with the ICC350 were the forced-coagulation mode at 20 W for marker dots; the endocut mode, effect3 at 100 W for mucosal incision; the forced-coagulation mode at 50 W for submucosal dissection; and the soft-coagulation mode at 80 W for hemostasis. The models used with the VIO300D were the soft-coagulation mode, effect 5 at 100 W for marker dots; the endocut I mode, effect 3, duration 3, interval 2 for mucosal incision; the swift-coagulation mode, effect 5, at 100 W for submucosal dissection; and the soft-coagulation mode, effect 5 at 100 W for hemostasis. For submucosal injection, saline, 20% concentrated glycerin-fructose (Glyceol; Chugai Pharmaceuticals, Tokyo, Japan), or 0.4% sodium hyaluronic acid (Mucoup; Johnson & Johnson, Tokyo, Japan) were used in ESD, either alone or mixed with 2% epinephrine (Bosmin; Daiichi Pharmaceuticals, Tokyo, Japan). The devices used to achieve hemostasis during ESD were hemostatic forceps (FD-410LR; Olympus or 1503; Boston Scientific, Boston, MA) for coagulating vessels. The day following ESD, scheduled second-look endoscopy was performed in about 98% of 1158 patients.

### 2.3. Assessments of Factors Associated with Delayed Perforation

The following clinicopathological factors were retrospectively analyzed by comparing cases with and without delayed perforation: sex (male versus female); age (<70 years versus ≥70 years), status of the stomach (normal/remnant versus stomach gastric tube), location (upper versus middle/lower), size (≤20 mm versus >20 mm), depth of invasion (M versus SM), ulceration (absent versus present), and procedure time (<2 h versus ≥2 h).

### 2.4. Definition of Delayed Perforation

Delayed perforation was defined as cases in which perforation had not been detected during and just after completion of ESD, but subsequent endoscopy showed perforation and computed tomography (CT) showed free air after ESD.

### 2.5. Statistical Analysis

On univariate analyses to assess clinicopathological factors, the *χ*^2^ test was used to compare cases with and without delayed perforation. The *t*-test was used to determine whether a significant difference in duration of cautery existed between perforated and nonperforated points.

## 3. Results

**Figure 1:**
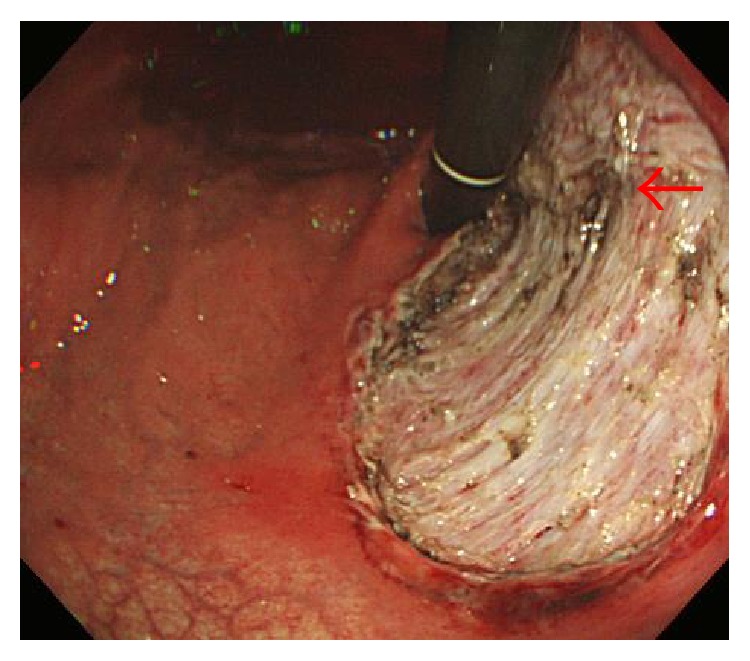
Case 1 underwent endoscopic submucosal dissection (ESD) for early gastric cancer in the upper third of the stomach. A vessel had been coagulated and cut (arrow). No perforation was observed just after completion of ESD.

**Figure 2:**
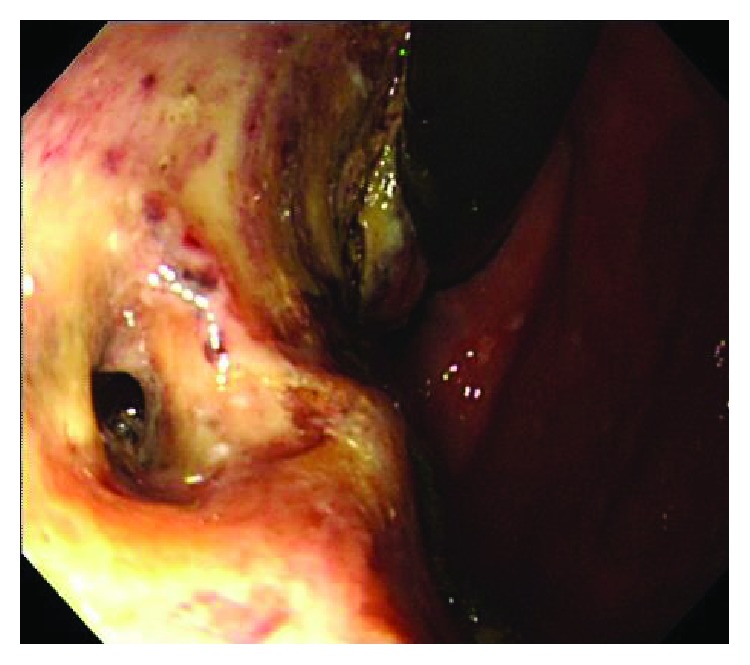
Delayed perforation occurred the day after ESD. Duration of cautery needed for hemostasis in this perforated points was a total of 11 s.

**Figure 3:**
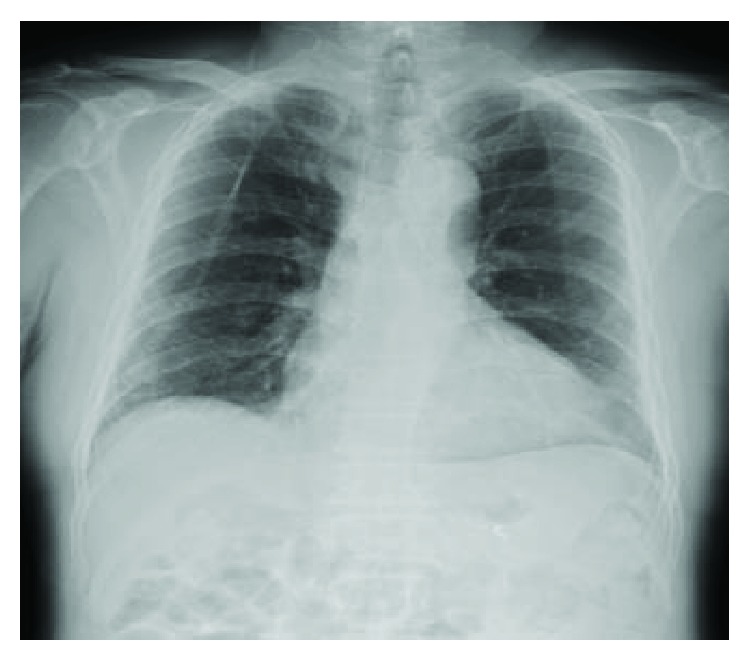
Chest X-ray examination in standing position does not reveal any free air.

**Figure 4:**
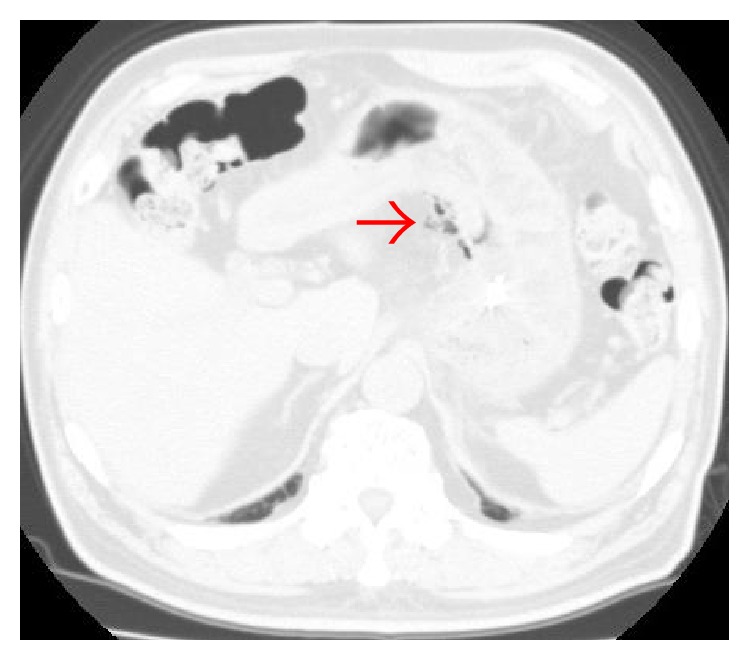
Computed tomography **(**CT) showed microfree air (arrow) in the omental bursa.

**Figure 5:**
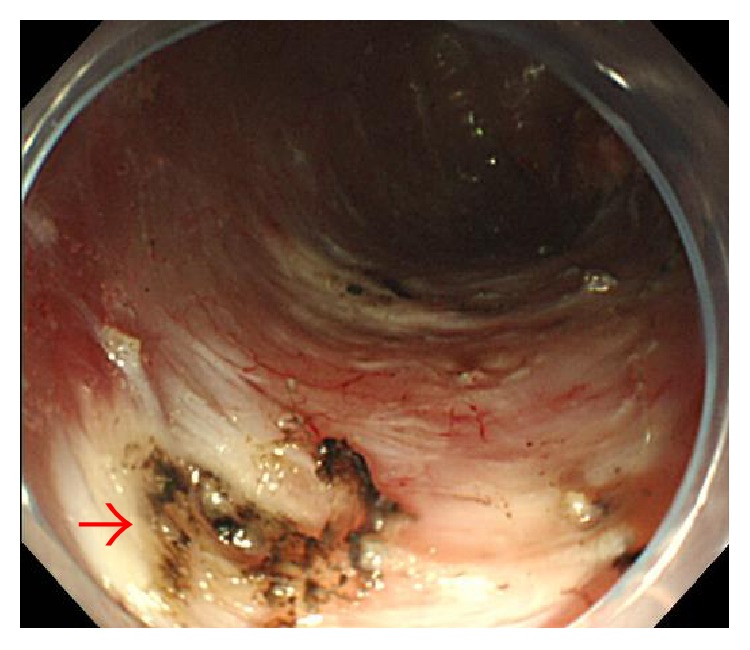
Case 5 underwent ESD for early gastric cancer in the lower third of the stomach. No perforation has been observed just after the completion of ESD. The arrow shows the perforated point on the next day after ESD.

**Figure 6:**
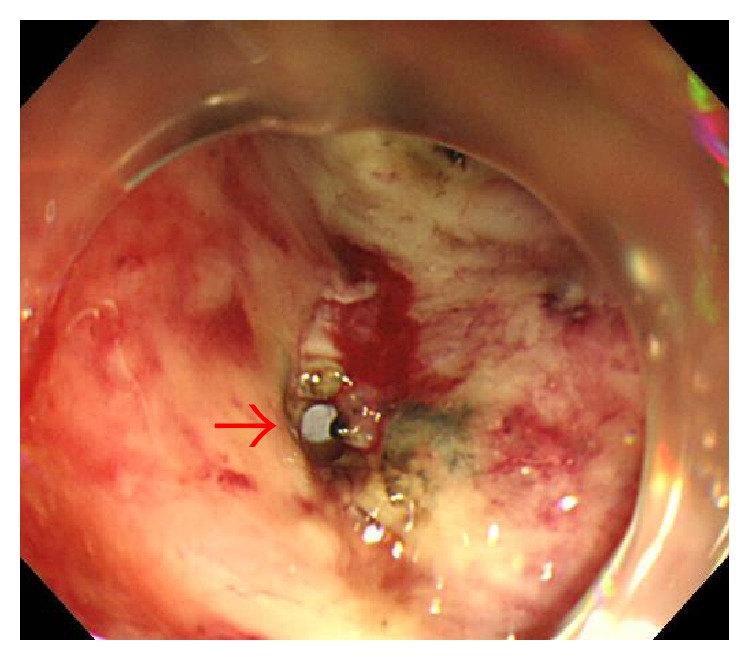
Delayed perforation occurred the day after ESD. Duration of cautery needed for hemostasis in this perforated points was a total of 7 s.

**Figure 7:**
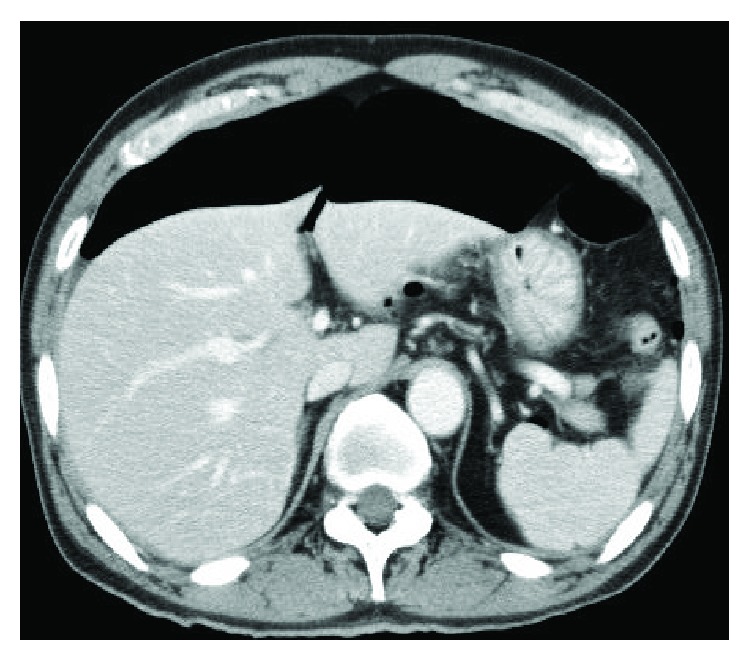
Emergent CT shows massive free air when the patient complained of severe abdominal pain.

Clinicopathological findings for the 1199 EGNs are shown (Table 1). Delayed perforation occurred in 5 of the 1199 EGNs that underwent ESD (0.42%). The clinicopathological features and clinical outcomes of patients with delayed perforation are shown in Table 2. Three patients were asymptomatic, and the delayed perforations were found on scheduled second-look gastroscopy (Figures 1, 2, 3, and 4). In the remaining two patients, emergent gastroscopy for symptoms of fever and abdominal pain showed delayed perforation (Figures 5, 6, and 7). All cases were diagnosed within 24 h after ESD. Median diameter of the delayed perforations observed by gastroscopy was 4 mm (range, 3–5). Four lesions were located in the upper third on the lesser curvature near the posterior wall of the stomach (Figure 1). One lesion was located in the lower third on the greater curvature of the stomach (Figure 5). In this study, delayed perforation may have been due to excessive electrocautery for hemostasis because of measurement of hemostatic duration with videos (Figures 2 and 5). Delayed perforation consistently occurred in the normal stomach and did not occur in postoperative stomach, such as a remnant stomach or gastric tube. The device used in the ESD procedures was the IT2 in all five cases of delayed perforation. Histological study of the resected specimens showed that all lesions represented differentiated adenocarcinoma, with no scar in the tumor. CT showed free air in all cases (Figures 4 and 7). Chest X-ray examinations in the standing position were performed in two cases and showed no free air (Figure 3). The two perforations without free air on chest X-ray examinations were located in the upper third on the lesser curvature near the posterior wall of the stomach. Endoscopic clips were successful for closing the perforations completely in four cases but proved unsuccessful in one case. In that unsuccessful case, surgical treatment was able to be avoided, because symptoms of peritonitis were not observed due to early detection of the perforation and administration of an antibiotic agent. As a result, all cases recovered with conservative management and did not require surgical treatment. Median time to oral intake was 7 days (range, 5–15 days). No local recurrences or distant metastases were observed during follow-up (median, 26 months; range, 8–31 months).

Clinicopathological factors were analyzed to identify associations with delayed perforation after gastric submucosal dissection (Table 3). Delayed perforation was more frequent in the upper third of the stomach (1.8%; 4/222) than in other regions (0.1%; 1/977). Based on univariate analyses, location in the upper third of the stomach was the only significant factor associated with delayed perforation (*P* < 0.01). Comparison of duration of cautery needed for hemostasis between perforated and nonperforated points is shown in Table 4. In the five patients, average duration of cautery needed for hemostasis at perforated points (9 s; *n* = 5) was significantly longer than that required at nonperforated points (3.5 s; *n* = 37). In addition, duration of cautery at perforated points was longest among all duration recorded for hemostasis of all bleeding points in each case.

## 4. Discussion

Delayed perforation after ESD for EGN is less common than perforation during the ESD procedure. Unlike perforation during ESD, delayed perforation is associated with a high rate of peritonitis and requires surgical treatment [10–14]. In the present study, however, all 5 cases recovered without surgical treatment. The early detection and diagnosis of delayed perforation before dietary intake allowed surgical treatment to be avoided. Rates of delayed perforation have been reported to range from 0.06% to 0.45% [3, 10–13]. The rate in the present study was similar, at 0.42% (5/1199). The size of delayed perforations reported in several previous cases ranged from 2 to 20 mm [10, 11, 13–18]. The mechanism underlying small, delayed perforation is considered to be excessive electrocautery for hemostasis [10, 11, 13–15, 17]. Large delayed perforations, such as around 20 mm in diameter, are thought to develop via different, as-yet unclear, mechanisms.

The present study showed that location in the upper third of the stomach was the factor most significantly associated with delayed perforation. Many studies have reported the upper-third location as associated with a higher rate of perforation during ESD [3, 6–8, 21]. In terms of bleeding, Oda et al. reported that rates of significant immediate bleeding are higher in the upper and middle thirds of the stomach than in the lower third because of the larger diameter of the submucosal arteries in the upper and middle thirds [3, 4, 22]. Excessive electrocautery for hemostasis in the upper third of the stomach during ESD was thus suggested to be associated with a higher rate of delayed perforation in the upper third location. Suzuki et al. reported that gastric tube cases were significantly associated with delayed perforation [10]. In the present study, delayed perforation did not occur in gastric tube cases.

Until now, no reports have shown a correlation between duration of cautery and delayed perforation. Also, duration of excessive electrocautery has been unclear. In this study, delayed perforation occurred with cautery lasting a total of 7 s in one patient. In another patient, delayed perforation occurred by cautery lasting 11 s. Average duration of cautery needed for hemostasis in the five patients at perforated points (*n* = 5) was 9 s, significantly longer than the 3.5 s needed at nonperforated points (*n* = 37). In addition, duration of cautery at perforated points was longest in duration for hemostasis among all bleeding points in each case. On the one hand, delayed perforation did not occur in 1194 of the 1199 EGNs that underwent ESD. Among 1194 cases without delayed perforation, 5 cases were selected randomly in order to examine average duration of cautery for hemostasis. The total number of points needed for hemostasis by cautery was 34 in 5 cases without delayed perforation, and average duration of cautery was also 3.5 s (data not shown). Based on our results, excessive electrocautery thus seems to represent a key risk factor for delayed perforation. This is the first report to show a statistical correlation between duration of cautery and delayed perforation.

The major symptoms of delayed perforation are abdominal pain and fever. However, more than half of cases showing delayed perforation in the present study were asymptomatic. Even if symptoms are absent due to the small size of the perforation in the posterior wall, dietary intake will inevitably give rise to peritonitis. Most cases of delayed perforation in previous reports were diagnosed within 1-2 days after ESD [10, 11, 13–15, 17, 18]. All 5 cases in the present study were discovered within 24 h after ESD on emergent or scheduled second-look gastroscopy. In three cases, scheduled second-look gastroscopy allowed the diagnosis of delayed perforation before peritonitis developed. Scheduled second-look gastroscopy after ESD has not been routinely recommended for the prevention of post-ESD bleeding [23]. However, in cases of delayed perforation, second-look gastroscopy is useful for the detection of perforation [15]. Therefore, in cases that need frequent electrocautery for hemostasis in the upper third of the stomach, second-look gastroscopy may be useful for early detection of delayed perforation even in asymptomatic patients.

Closing a perforation with endoclips is easier on the day of ESD for EGN [24]. Closing a perforation with endoclips several days after ESD would be more difficult, because of necrosis around the perforation [18]. Some cases of conservative treatment for delayed perforations have been reported [10, 11, 15–18]. Surgical treatment can be avoided if the perforation is small and the diagnosis is reached early, before oral intake is resumed, at the very least. Surgical treatment is inevitable if the perforation is found with progressive peritonitis or if the perforation is large. In this report, early detection and management of delayed perforation allowed us to avoid surgical treatment. In one patient, endoclips failed to close the perforation, but surgical treatment was avoided due to delayed dietary intake for 15 days.

Suitable preventive measures for delayed perforation have not yet been established. However, two measures are applied for the prevention of delayed perforation in our hospital. First, endoclips are applied to vessels requiring excessive electrocautery (about 9 s of duration) for hemostasis. Second, in addition to endoclips, a nasogastric (NG) tube is inserted into the stomach to reduce intrastomach pressure. The NG tube makes it possible to create negative pressure within the stomach using medical equipment or a manual method. Use of an NG tube is limited to those cases considered to be at high risk of delayed perforation. With such measures, no instances of delayed perforation have occurred in the last 3 years.

The present study had several limitations. First, this was a retrospective study of our own database and medical records of patients with consecutive gastric ESD for EGNs. Second, the study cohort was small, because the number of delayed perforations was small. Third, this study was conducted at a single center, and gastric ESD procedures were mainly performed by five highly experienced endoscopists. A multicenter prospective cohort study of ESD for early gastric cancer is currently underway [25].

## 5. Conclusions

In conclusion, lesion in the upper third of the stomach was significantly associated with delayed perforation. Moreover, excessive duration of cautery correlated with delayed perforation.

## Figures and Tables

**Table 1:** Clinicopathological findings of 1199 early gastric neoplasms undergoing endoscopic submucosal dissection.

Clinicopathological findings	*n* (%)
Sex	
Male	937 (78.1)
Female	262 (21.9)
Age	
Median (range)	71 (41–92)
<70	524 (43.7)
≥70	675 (56.3)
Stomach status	
Normal stomach	1151 (96.0)
Remnant stomach	33 (2.7)
Gastric tube	15 (1.3)
Location	
Upper	222 (18.5)
Middle	510 (42.5)
Lower	467 (39.0)
Size (mm)	
Median (range)	14 (1–73)
≤20	893 (74.5)
>20	306 (25.5)
Depth of invasion	
M	1029 (85.8)
SM	170 (14.2)
Ulceration	
Absent	1051 (87.7)
Present	148 (12.3)
Procedure time (hours)	
<2	863 (71.8)
≥2	336 (28.2)

M: mucosa; SM: submucosa.

**Table 2:** Clinicopathological features and clinical outcomes of 5 patients with delayed perforation after endoscopic submucosal dissection (ESD) for early gastric neoplasms.

Case number	Age	Sex	Tumor location	Tumor size (mm)	Depth of tumor	Time required for ESD (minutes)	Time until diagnosis (hours)	Symptoms	Free air on X-ray	Free air on CT	Size of delayed perforation (mm)	Endoclips	Treatment	Time to oral intake (days)	Hospital stay (days)
1	67	Male	U Pos	22	Submucosa	180	17	None	Negative	Positive	4	Successful	Conservative	8	13
2	79	Female	U Les	23	Mucosa	120	19	None	Negative	Positive	4	Successful	Conservative	6	14
3	80	Female	U Pos	15	Mucosa	55	8	Fever and pain	NE	Positive	3	Successful	Conservative	5	12
4	76	Male	U Les	54	Submucosa	85	21	None	NE	Positive	5	Unsuccessful	Conservative	15	22
5	72	Male	L Gre	20	Mucosa	35	14	Fever and pain	NE	Positive	3	Successful	Conservative	7	15

U: upper third; L: lower third; Les: lesser curvature; Gre: greater curvature; NE: not evaluated; CT: computed tomography.

**Table 3:** Clinical factors related to delayed perforation after gastric endoscopic submucosal dissection (ESD) *n* (%).

Clinicopathological finding	Univariate analysis		
	Cases without delayed perforation *n* = 1194 (99.58)	Cases with delayed perforation *n* = 5 (0.42)	*P* value
Sex			0.32
Male	934 (99.7)	3 (0.3)	
Female	260 (99.2)	2 (0.8)	
Age (yr)			0.28
<70	523 (99.8)	1 (0.2)	
≥70	671 (99.4)	4 (0.6)	
Stomach status			0.8
Normal/remnant stomach	1179 (99.6)	5 (0.4)	
Gastric tube	15 (100)	0 (0.0)	
Location			0.0004
Upper	218 (98.2)	4 (1.8)	
Middle/lower	976 (99.9)	1 (0.1)	
Size (mm)			0.076
≤20	891 (99.8)	2 (0.2)	
>20	303 (99.0)	3 (1.0)	
Depth of invasion			0.097
M	1026 (99.7)	3 (0.3)	
SM	168 (98.8)	2 (1.2)	
Ulceration			0.4
Absent	1046 (99.5)	5 (0.5)	
Present	148 (0.0)	0 (0.0)	
Procedure time (hours)			0.55
<2	863 (99.7)	3 (0.3)	
≥2	334 (99.4)	2 (0.6)	

**Table 4:** Total duration of cautery needed for hemostasis by comparison between perforated points and nonperforated points in five cases.

	Perforation (*n* = 5) average (range)	No perforation (*n*^∗^ = 37) average (range)	*P* value
Duration (second)	9 (7–11)	3.5 (2–8)	*P* < 0.001

*n* ^∗^: the number of points needed for hemostasis without perforation in five cases.

## References

[1] Gotoda T., Yanagisawa A., Sasako M. (2000). Incidence of lymph node metastasis from early gastric cancer: estimation with a large number of cases at two large centers. *Gastric Cancer*.

[2] Kim Y. J., Park D. K. (2011). Management of complications following endoscopic submucosal dissection for gastric cancer. *World Journal Gastrointestinal Endoscopy*.

[3] Oda I., Suzuki H., Nonaka S., Yoshinaga S. (2013). Complications of gastric endoscopic submucosal dissection. *Digestive Endoscopy*.

[4] Miyahara K., Iwakiri R., Shimoda R. (2012). Perforation and postoperative bleeding of endoscopic submucosal dissection in gastric tumors: analysis of 1190 lesions in low- and high-volume centers in Saga, Japan. *Digestion*.

[5] Toyokawa T., Inaba T., Omote S. (2012). Risk factors for perforation and delayed bleeding associated with endoscopic submucosal dissection for early gastric neoplasms: analysis of 1123 lesions. *Journal of Gastroenterology and Hepatology*.

[6] Ohta T., Ishihara R., Uedo N. (2012). Factors predicting perforation during endoscopic submucosal dissection for gastric cancer. *Gastrointestinal Endoscopy*.

[7] Yoo J. H., Shin S. J., Lee K. M. (2012). Risk factors for perforations associated with endoscopic submucosal dissection in gastric lesions: emphasis on perforation type. *Surgical Endoscopy*.

[8] Kim M., Jeon S. W., Cho K. B. (2013). Predictive risk factors of perforation in gastric endoscopic submucosal dissection for early gastric cancer: a large, multicenter study. *Surgical Endoscopy*.

[9] Watari J., Tomita T., Ikehara H. (2015). Diagnosis of small intramucosal signet ring cell carcinoma of the stomach by non-magnifying narrow-band imaging: a pilot study. *World Journal Gastrointestinal Endoscopy*.

[10] Suzuki H., Oda I., Sekiguchi M. (2015). Management and associated factors of delayed perforation after gastric endoscopic submucosal dissection. *World Journal of Gastroenterology*.

[11] Hanaoka N., Uedo N., Ishihara R. (2010). Clinical features and outcomes of delayed perforation after endoscopic submucosal dissection for early gastric cancer. *Endoscopy*.

[12] Kato M., Nishida T., Tsutsui S. (2011). Endoscopic submucosal dissection as a treatment for gastric noninvasive neoplasia: a multicenter study by Osaka University ESD Study Group. *Journal of Gastroenterology*.

[13] Yano T., Tanabe S., Ishido K. (2016). Delayed perforation after endoscopic submucosal dissection for early gastric cancer: clinical features and treatment. *World Journal Gastrointestinal Endoscopy*.

[14] Kang S. H., Lee K., Lee H. W., Park G. E., Hong Y. S., Min B. H. (2015). Delayed perforation occurring after endoscopic submucosal dissection for early gastric cancer. *Clinical Endoscopy*.

[15] Ikezawa K., Michida T., Iwahashi K. (2012). Delayed perforation occurring after endoscopic submucosal dissection for early gastric cancer. *Gastric Cancer*.

[16] Onozato Y., Iizuka H., Sagawa T. (2006). A case report of delayed perforation due to endoscopic submucosal dissection (ESD) for early gastric cancer. *Progress of Digestive Endoscopy*.

[17] Hirasawa T., Yamamoto Y., Okada K. (2009). A case of the delayed perforation due to endoscopic submucosal dissection for the early gastric cancer of the residual stomach. *Progress of Digestive Endoscopy*.

[18] Ono H., Takizawa K., Kakushima N., Tanaka M., Kawata N. (2015). Application of polyglycolic acid sheets for delayed perforation after endoscopic submucosal dissection of early gastric cancer. *Endoscopy*.

[19] Japanese Gastric Cancer Association (2011). Japanese gastric cancer treatment guidelines 2010 (ver. 3). *Gastric Cancer*.

[20] Ono H., Hasuike N., Inui T. (2008). Usefulness of a novel electrosurgical knife, the insulation-tipped diathermic knife-2, for endoscopic submucosal dissection of early gastric cancer. *Gastric Cancer*.

[21] Yoon J. Y., Shim C. N., Chung S. H. (2014). Impact of tumor location on clinical outcomes of gastric endoscopic submucosal dissection. *World Journal of Gastroenterology*.

[22] Kikuchi D., Iizuka T., Hoteya S. (2013). Prospective study about the utility of endoscopic ultrasound for predicting the safety of endoscopic submucosal dissection in early gastric cancer (T-HOPE 0801). *Gastroenterology Research and Practice*.

[23] Mochizuki S., Uedo N., Oda I. (2015). Scheduled second-look endoscopy is not recommended after endoscopic submucosal dissection for gastric neoplasms (the SAFE trial): a multicentre prospective randomised controlled non-inferiority trial. *Gut*.

[24] Minami S., Gotoda T., Ono H., Oda I., Hamanaka H. (2006). Complete endoscopic closure of gastric perforation induced by endoscopic resection of early gastric cancer using endoclips can prevent surgery (with video). *Gastrointestinal Endoscopy*.

[25] Oda I., Shimazu T., Ono H. (2012). Design of Japanese multicenter prospective cohort study of endoscopic resection for early gastric cancer using Web registry (J-WEB/EGC). *Gastric Cancer*.

